# The pollen tube's secret to slick growth? A dab of pectate lyase-like enzyme

**DOI:** 10.1093/plphys/kiad522

**Published:** 2023-10-03

**Authors:** Janlo M Robil

**Affiliations:** Assistant Features Editor, Plant Physiology, American Society of Plant Biologists; Department of Biology, School of Science and Engineering, Ateneo de Manila University, Quezon City 1108, Philippines

In flowering plants, the nonmotile sperm are carried to the egg by a tube that emerges from the pollen grain. This process begins after pollination, when the pollen tube rapidly elongates to find the egg-bearing ovule within the pistil (Fig. 1A). The directional growth of the pollen tube is driven by turgor pressure and sustained by the expansion of the cell wall at the tip (Chebli and Geitmann 2007). Pollen tube elongation thus illustrates a delicate balance between growth mechanics and cell wall dynamics, making it a fantastic model for apical cell growth in plants.

**Figure 1.**
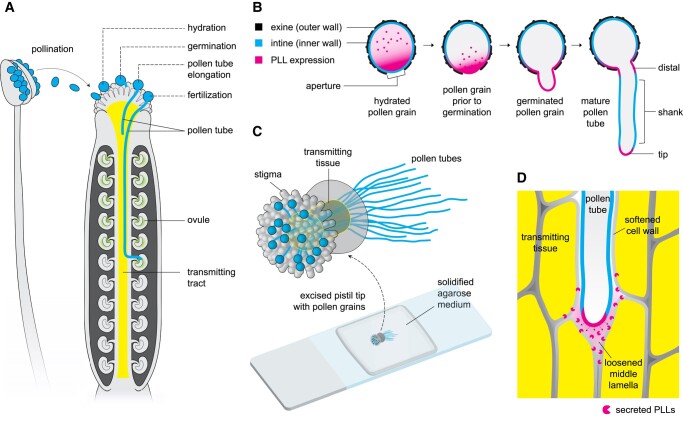
The path of the pollen tube toward fertilization and the roles of PLL enzymes. **A)** Schematic diagram of Arabidopsis anther and pistil showing the stages of interaction of the pollen grain/tube with the pistil. **B)** Schematic diagram of the expression patterns of PLLs from pollen grain hydration to pollen tube elongation. Localization and concentration of PLLs are indicated by magenta color. **C)** An illustration of semi-in vivo pollen tube growth where pollen grains are germinated on the stigma of an excised pistil. The pollen tubes penetrate the transmitting tissue and continue to grow on the agarose medium. **D)** Schematic diagram of pollen tube's invasion of the transmitting tissue. Chebli and Geitmann (2023) propose that PLLs are secreted by the pollen tube into the extracellular environment to aid in the lubrication of its growth path, contributing to the successful fertilization.

To deliver its cargo, the pollen tube must not only elongate but also overcome obstacles along its growth path, the transmitting tract. Some plants, like lilies, have hollow transmitting tracts, whereas others, including Arabidopsis, have solid or semi-solid tracts. In the latter, the pollen tube must either induce programmed cell death or digest the apoplast to penetrate the tissue (Chebli and Geitmann 2007). Therefore, the pollen tube's ability to grow both expansively and invasively is critical for its function in the fertilization process.

The pollen tube is known to produce cell wall–modifying enzymes to aid its growth (Jiang et al. 2005; Chebli and Geitmann 2007; Chebli et al. 2012). However, until now, there has been no experimental proof that it also secretes enzymes to “lubricate” its own path during elongation. In this issue of *Plant Physiology*, Chebli and Geitmann (2023) tested that idea and explored the role of a pectin-digesting enzyme, pectate lyase-like (PLL), in Arabidopsis. They examined knock-out mutants and GFP-tagged transgenic lines and used fluorescence recovery and protein detection assays to confirm that pollen tubes release PLLs, which may digest the transmitting tissue. Their work reveals the key roles of PLLs during pollen tube emergence, growth, and invasion of the transmission tract.

After landing on the stigma, a compatible pollen grain is hydrated, leading to the emergence of the pollen tube. Pollen grains have 2 layers of walls: an extremely resistant outer wall and an inner wall that expands to form the pollen tube andthat is primarily made of cellulose and pectin (Fig. 1B). PLLs have been hypothesized to relax the pollen grain's inner wall, enabling it to protrude through the outer wall's aperture. Chebli and Geitmann (2023) showed that PLLs are abundant in hydrated pollen grains and that they are specifically localized at the aperture during germination (Fig. 1B). These findings suggest that PLLs may play a role during pollen tube emergence, which is consistent with the drastic reduction of pollen germination in single *pll* knock-out mutants.

Upon successful germination, the pollen tube then grows to penetrate the stigma and the transmitting tract (Fig. 1A). The authors found that *PLLs* (*PLL1–3*) are expressed at the distal region close to the aperture and the very tip of the growing pollen tube, but *PLL3* is also expressed in specific regions along the shank (Fig. 1B). In semi*-*in vivo culture (Fig. 1C), the authors also found that single *pll* mutants have significantly delayed pollen tube elongation during the early stages of growth. This phenotype may have resulted in altered progeny ratios when attempting to generate double *pll* mutants. As no double homozygotes were recovered, the authors deduced that pollen grains with 2 mutant alleles were most likely outcompeted by the other genotypes during fertilization, consistent with the observed progeny ratios. These findings indicate that PLLs are essential for pollen tube elongation and suggest a possible subfunctionalization of *PLL3*.

During elongation, the pollen tube invades the transmitting tissue. It has long been theorized that the pollen tube releases cell wall– or matrix-digesting enzymes to facilitate this process (Heslop-Harrison 1987). Although several of these enzymes are known to be produced by the pollen tube, it is not yet known whether they are actually secreted into the tissue.

In a series of experiments, Chebli and Geitmann (2023) found evidence suggesting that PLLs are secreted by the pollen tube. First, they found that PLLs were not localized in the plasma membrane but rather in the extracellular space, as observed in plasmolyzed cells. This finding suggests that PLLs are highly soluble, which agrees with the predicted protein models. Next, the authors performed fluorescence recovery after photobleaching experiments and discovered that newly synthesized PLLs are consistently shuttled to the tip of the pollen tube. The proteins were neither recycled nor accumulated in the wall, suggesting their secretion into the extracellular environment. Finally, the authors detected PLLs in the in vitro growth medium, which supports the findings of the fluorescence recovery after photobleaching experiments. Collectively, the localization of PLLs in the extracellular space, their intracellular mobility, and their presence in the growth medium point to the secretion of the protein by the pollen tube.

The work of Chebli and Geitmann (2023) revealed previously unknown roles of PLLs in pollen tube germination and elongation, which posit interesting questions for future research. For example: Precisely how are PLLs activated at the aperture during pollen germination? How does the subfunctionalization in *PLLs* contribute to the control of biomechanics and cell wall dynamics in the pollen tube and other tip-growing cells? And what roles do PLLs play in directional pollen tube growth and other responsive polarized cell growth (Burri et al. 2018)?

In addition, Chebli and Geitmann (2023) addressed the long-standing question of whether the pollen tube participates in softening the transmitting tract. They concluded that the pollen tube secretes PLLs, proposing that these enzymes aid in its invasion of the transmitting tissue (Fig. 1). For example, it is possible that the digestion of pectin in the transmitting tissue apoplast could create a liquefied, lubricated pathway for the growing pollen tube. Although it remains uncertain whether secreted PLLs digest pectin *in planta*, this work prompts questions about the downstream effects of such enzymatic action. One question is whether digested pectin acts as signaling molecules for pollen tube guidance or perception. Another question is whether the products of digestion are consumed and utilized by the growing pollen tube.

As unpredictable climates make it increasingly challenging to ensure successful fertilization in agricultural crops (Kim et al. 2019), the work by Chebli and Geitmann (2023) could have far-reaching agricultural implications if PLLs or similar enzymes can be used to increase fertilization efficiency or overcome hybridization barriers. Therefore, this work lays the foundation for future research on exciting and important aspects of pollen-pistil interaction in flowering plants.

## Dive Curated Terms

The following phenotypic, genotypic, and functional terms are of significance to the work described in this paper:

pollen AmiGo: PO:0025281
